# Spectrophotometric Study of Erbium(III) Speciation in Chloride Solutions from 25 to 75 °C

**DOI:** 10.1021/acsearthspacechem.6c00161

**Published:** 2026-08-31

**Authors:** Hannah Juan Han, Alexander P. Gysi, Nicole C. Hurtig, Artas A. Migdisov

**Affiliations:** † New Mexico Bureau of Geology and Mineral Resources, 7374 New Mexico Institute of Mining and Technology, 801 Leroy Place, Socorro, New Mexico 87801, United States; ‡ Department of Earth and Environmental Science, New Mexico Institute of Mining and Technology, 801 Leroy Place, Socorro, New Mexico 87801, United States; § McGill University, Earth&Environ. Sciences, Montréal H3A 0G4, Canada

**Keywords:** rare earth elements, UV−vis spectrophotometry, erbium, chloride, formation constant, speciation

## Abstract

Accurate thermodynamic properties for REE aqueous complexes are crucial for predicting the mobility of rare earth elements (REE) in natural waters and for developing improved separation and recovery technologies. In this study, UV–vis spectrophotometric experiments were conducted at 25–75 °C using pH 1.5 of Er-HClO_4_-bearing solutions with varying NaCl concentrations from 0 to 1.7 mol/kg. Under these experimental conditions, Er hydrolysis is negligible, and speciation is controlled by Er^3+^ and Er chloride complexes. The molar absorbance coefficient of Er^3+^ decreases with temperature, and the absorbance intensity gradually decreases with increasing m_Cl_/m_Er_ ratios. The number of absorbing species include Er^3+^ and ErCl^2+^ even at the highest m_Cl_/m_Er_ ratio of ∼34. The formation constant (β_1_^0^) for the ErCl^2+^ species was derived and fitted between 50 and 250 °C with existing literature values, yielding the following equation: logβ_1_^0^ = −22.4315 + 0.0345*T* + 3.9379 × 10^3^/*T*, where *T* is temperature in Kelvin. A comparison between this new fit and previous predictions indicates good agreement at 100–250 °C, whereas larger differences occur in the 25–75 °C temperature range due to a broad scattering of previous experimental literature data at 25 °C. The new fit shows a systematic decrease in logβ_1_^0^ values from 25 to 100 °C, indicating that REE chloride complexes become less stable with increasing temperature. These updated formation constant data are important for modeling REE speciation at low temperatures relevant to REE sorption onto clay minerals and REE extraction technologies.

## Graphical abstract

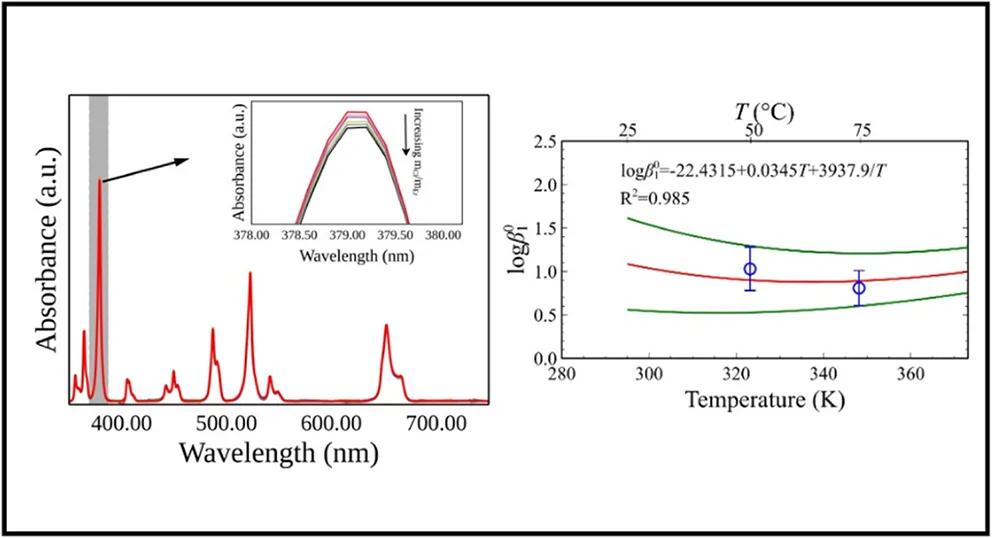

## 1 Introduction

Rare earth elements (REE) are essential components for high-tech and green technologies due to their unique optical, magnetic, and catalytic properties.^1–3^ The growing demand for REE has intensified the need to better understand the behavior of aqueous REE species in natural systems and to develop more efficient separation and extraction technologies. Thermodynamic modeling and experimental studies indicate that hydrothermal NaCl-bearing aqueous solutions play a key role in controlling REE mobility during the formation of critical mineral ore deposits.^4–10^ The stability of REE chloride complexes can significantly influence REE mobility at low pH, whereas at high pH, the REE become more immobile due to the formation of hydroxyl complexes and the resulting decrease in REE mineral solubilities.^11–14^

The stability of aqueous Er chloride complexes (ErCl^2+^ and ErCl_2_^+^) has been investigated using various experimental methods, including solubility measurements, UV–vis spectrophotometry, potentiometric measurements, and solvent extraction measurements. Mironov^15^ performed solubility experiments at 25 °C and reported a logarithmic value of the first formation constant (logβ_1_^0^) of 0.26. Luo and Byrne^16^ conducted potentiometric experiments at 25 °C and ionic strengths of 0.7, 1.5, 3, 4, and 5 mol/kg and derived a logβ_1_^0^ value of 0.71 ± 0.05. Fernández-Ramírez et al.^17^ carried out solvent extraction experiments at 30 °C in 2, 3, and 4 mol/L HCl/HClO_4_ solutions and reported a logβ_1_^0^ value to be 1.20 ± 0.5. Soderholm et al.^18^ performed high-energy X-ray scattering analysis and determined a logβ_1_ value of −0.41 in a 4.0 mol/kg HClO_4_ solution. The logβ_1_^0^ values of ErCl^2+^ display about an order-of-magnitude scattering based on experimental literature data compiled^15,16^ at 25 °C. Similar discrepancies have recently been addressed for other complexes, such as the Er hydroxyl complexes, using UV–vis spectrophotometry.^11,12^

Migdisov and Williams-Jones^19^ studied Er chloride complexes between 100 and 250 °C using UV–vis spectrophotometry. Their experiments indicate that logβ_1_^0^ values for ErCl^2+^ increase from 0.88 ± 0.11 to 3.09 ± 0.14 with increasing temperature. They also report logβ_2_^0^ values for ErCl_2_^+^ of 2.95 ± 0.34 and 4.12 ± 0.12 at 200 and 250 °C, respectively. Migdisov et al.^20^ also report logβ_1_^0^ and logβ_2_^0^ values for REE chloride complexes from 25 to 300 °C based on solubility experiments and derived a set of Helgeson–Kirkham–Flowers (HKF) equation-of-state model parameters for geochemical modeling of REE chloride speciation. The logβ_1_^0^ and logβ_2_^0^ values for Er chloride complexes increase significantly with temperature, from 0.70 and 0.45 at 25 °C to 3.96 and 5.55 at 300 °C. Gammon et al.^21^ performed solubility experiments in 1 mol/kg silver chloride and derived a logβ_1_^0^ value of 2.17 at 200 °C. A recent study by Kershaw et al.^22^ reports the logβ_1_^0^ and logβ_2_^0^ values for ErCl^2+^ and ErCl_2_^+^ complexes at supercritical conditions, showing even higher logβ_1_^0^ values of 2.6 and 8.1, and logβ_2_^0^ of 5.0 and 17.8, at 350 to 450 °C, respectively. This indicates a substantial increase in the stability of Er chloride complexes at supercritical conditions. Despite previous experimental efforts, there are still significant gaps in experimental data between 25 and 100 °C, which are important to address modeling model low temperature REE mobility in natural waters and for the development of new potential REE recovery and separation technologies.

In this study, UV–vis spectrophotometric experiments were conducted at acidic pH and at temperatures between 25 and 75 °C. We determined the absorbance coefficients for Er species, the number of contributing species (i.e., Er^3+^ and Er chloride), and the formation constants for Er chloride complexes. These new experimental data are fitted together with existing literature data to provide improved formation constants from 25 to 250 °C.

## 2 Materials and Methods

### 2.1 Materials

The experimental NaCl-bearing Er_2_O_3_–HClO_4_ solutions with a pH of 1.5 were prepared from the stock solutions to achieve final concentrations of ∼0.06 mol/kg Er and varying NaCl concentrations ranging from 0 to 1.7 mol/kg. An HClO_4_ stock solution was prepared by using Milli-Q water (18.2 MΩ·cm) and concentrated perchloric acid (Fisher Scientific, trace metal grade) to a final pH of 1.5. An Er_2_O_3_–HClO_4_ stock solution was prepared by dissolving solid Er_2_O_3_ (Alfa Aesar, Reacton grade, 99.99% pure) in Milli-Q water and acidified by concentrated perchloric acid to a final pH of 1.5. This Er stock solution was subsequently diluted using a pH of 1.5 HClO_4_ stock solution to generate ∼0.06 mol/kg Er. The NaCl-bearing stock solution was prepared by dissolving high-purity sodium chloride (Thermo Scientific Puratronic, 99.998% metal basis) in the prepared Er_2_O_3_–HClO_4_ stock solution to a concentration of ∼1.7 mol/kg NaCl and ∼0.06 mol/kg Er. This solution was subsequently diluted using the Er_2_O_3_–HClO_4_ stock solution to achieve a range of NaCl concentrations from ∼0.01 to 1.7 mol/kg.

### 2.2 UV–vis Experiments

Spectrophotometric measurements were conducted at temperatures from 25 to 75 °C in NaCl-free and NaCl-bearing Er_2_O_3_–HClO_4_ solutions at wavelengths from 350 to 750 nm at 0.2 nm intervals using an Agilent Cary 4000 dual-beam UV–vis spectrophotometer in the Ore Deposits and Critical Minerals Laboratory, at the New Mexico Bureau of Geology and Mineral Resources (NMBGMR), New Mexico Institute of Mining and Technology. The wavelength accuracy of the instrument was calibrated and verified using the alpha (656.1 nm) and beta (486 nm) emission lines of the deuterium lamp. The spectral bandwidth of the instrument is 1.0 nm. An ∼3 mL aliquot of the experimental solutions was loaded in 10 mm path-length rectangular quartz cells sealed with Teflon. The temperature was maintained using an Agilent dual-cell Peltier temperature-control accessory mounted in the UV–vis instrument. Temperature was recorded using a Cary temperature probe with an accuracy of ±0.1 °C. An experimental estimate of uncertainty of the measured absorbance value at each isotherm was obtained through triplicate spectral measurements of a NaCl-free HClO_4_ solution containing a total Er concentration of 0.06 mol/kg. In this study, no unexpected or unusually high safety hazards were encountered.

### 2.3 Analytical Methods

The pH of the HClO_4_ stock solution and Er_2_O_3_–HClO_4_ solution was measured using a 913 Metrohm pH meter (accuracy of ±0.003) equipped with a pH electrode (Metrohm 6.0260.010 Unitrode with Pt1000 temperature sensor) calibrated using 1.68, 4.01, and 7.00 Fisher buffer solutions (accuracy of ± 0.01). Dissolved Na and Er concentrations in the prepared pH 1.5 of NaCl-free and NaCl-bearing Er_2_O_3_–HClO_4_ stock solutions were measured by using an Agilent 5900 inductively coupled plasma optical emission spectrophotometer (ICP-OES) at the Analytical Chemistry Laboratory, NMBGMR. A trace metal grade 1000 ± 5 ppm indium stock solution (Inorganic Ventures) was diluted with 2% HNO_3_ blank to 10 ppm as an internal standard for drift correction in the ICP-OES measurements. A series of Er analytical calibration standards in a concentration ranging from 25 to 1000 ppb was prepared from a 10 ± 0.04 ppm multi-REE stock standard (Inorganic Ventures, NIST traceable standard) diluted with a 2% HNO_3_ (Fisher Scientific, trace metal grade) blank matrix solution for the ICP-OES analysis. The Er analytical precision in the diluted ∼380 ppb Er_2_O_3_–HClO_4_ stock solution is better than 1.5%. The instrumental detection limit of Er is 1 ppb based on repeated analysis of the blank (5σ).^23^ A series of Na standards ranging from 0.5 to 10 ppm was prepared by using a Na single-element standard solution (Inorganic Ventures, CGNA1, 1002 ± 4 ppm) diluted with the 2% HNO_3_ blank. The Na concentration measured on ICP-OES has an analytical precision better than 2% based on repeated standard analysis at 1.7 ppm of Na. The limit of detection for Na is 10 ppb using repeated analysis of the blank (5σ).

## 3 Data Treatment

### 3.1 Determination of Absorbing Species

The absorbing species in NaCl-bearing Er_2_O_3_–HClO_4_ solutions at 25–75 °C are determined by using the collected UV–vis spectra and the Lambert–Beer law as described below:

1 $$\frac{A}{L}=\underset{i}{\sum }\varepsilon_{i}\times C_{i}$$

where *A* is the absorbance; ε_*i*_ is the molar absorbance of the corresponding species *i*; *C*_*i*_ is the molar concentration of the corresponding species, and L is the path length in cm, here constant of 1 cm. In NaCl-free Er_2_O_3_–HClO_4_ solutions, where only Er^3+^ aqua ions are present, the molar absorbance coefficient of Er^3+^ ions is determined using eq 1.

Addition of varying concentrations of NaCl to the Er_2_O_3_–HClO_4_ solutions results in the possible presence of additional Er chloride complexes, i.e., ErCl^2+^ and ErCl_2_^+^ that can contribute to the measured absorbance. Conversely, increasing NaCl concentration results in a decrease in the free Er^3+^ concentration and thus its absorbance. The number of absorbing species at each temperature was determined by evaluating the rank of an absorbance matrix, following the approach described by Suleimenov and Seward^24^ and Varga and Veatch.^25^ Under an assumption of a linear relationship between chemical composition and absorbance (eq 1), the number of absorbing species contributing to the matrix corresponds to the number of linearly independent vectors. The rank of the matrix, i.e., number of absorbing species, is calculated using a MATLAB program based on the singular value decomposition method according to

2 $$A=USV^{T}$$

where U and V are matrices with orthogonal columns and S is the diagonal matrix of singular values. This approach has been widely applied in UV–vis spectrophotometry studies to determine the number of absorbing species present in solutions.^19,26–31^ Considering that the complexes involving hydroxide are unlikely to be important at a pH of 1.5, the probable absorbing species for the Er species are Er^3+^, ErCl^2+^, and ErCl_2_^+^. Other species present in the background solutions include H^+^, OH^–^, H_2_O, Na^+^, NaCl^0^, HCl^0^, Cl^–^, and ClO_4_^–^.

### 3.2 Cumulative Formation Constants of Aqueous Er Chloride Complexes

The cumulative formation constants (β_n_^0^) of Er chloride complexes according to eq 3 at infinite dilution in NaCl-bearing Er_2_O_3_–HClO_4_ solutions at 25–75 °C are described as follows:

3 $$Er^{3+}+nCl^{-}=ErCl_{n}^{3-n}$$

4 $$\beta_{n}^{o}=\frac{\left[ErCl_{n}^{3-n}\right]}{\left[Er^{3+}\right]{[Cl^{-}]}^{n}}\times \frac{\gamma_{ErCl_{n}^{3-n}}}{\gamma_{Er^{3+}}\gamma_{Cl^{-}}^{n}}$$

where *n* is the number of chloride ligands bound to Er^3+^ aqua ions, with an expected value of 1 under the investigated conditions; [ErCl_n_^3‑n^], [Er^3+^], and [Cl^–^] are the molarities, and γ_ErCl*n* ^3–^ *^n^*_, γ_Er^3+^_, and γ_Cl^–^_ are the activity coefficients of the respective species.

The formation constants of the Er chloride complexes under the investigated conditions were determined from the measured UV–vis absorbance for each solution using the method described in previous studies.^19,24,26,27,29–31^ In this approach, the formation constant is obtained iteratively, starting from an initial estimate of the formation constants and refined through successive minimization of the following function:

5 $$U=\sqrt{\overset{J}{\underset{i=1}{\sum }}\overset{K}{\underset{k=1}{\sum }}{\left(\frac{A_{ik}^{\exp}-A_{ik}^{\mathrm{calc}}}{A_{ik}^{\exp}}\right)}^{2}}$$

where *i* is the wavelength; *J* is the total number of wavelengths collected; *K* is the number of solutions; *A*_*ik*_^exp^ is the measured absorbance using UV–vis; and *A*_*ik*_^calc^ is the calculated absorbance that depends on the absorbing species’ concentrations and their molar absorbance coefficients. The minimization procedure followed the algorithm of Nelder and Mead,^32^ as implemented in the MATLAB function library. The calculations were carried out through multiple iterative cycles aimed at minimizing the function U (eq 5) with respect to the formation constant. In each iteration, the concentrations of Cl^–^, Er^3+^, ErCl^2+^, and ErCl_2_^+^ species in each experimental solution were calculated from the total concentrations of Er and Cl, together with initial estimates of the formation constants. These calculated Er concentrations were subsequently used to deconvolute the absorbance matrix, yielding optimized values of molar absorbance coefficients at each experimental wavelength. The activity coefficients (γ_*i*_) of charged aqueous species are calculated using the extended Debye–Hückel equation as follows:^33^

6 $$\log\gamma i=\frac{-Az_{i}^{2}\sqrt{I}}{1+åB\sqrt{I}}+b_{\gamma }I$$

and the effective ionic strength (*I*) is given by

7 $$I=1/2\sum m_{i}z_{i}^{2}$$

where *A* and *B* are the Debye–Hückel parameters;^34,35^ *b*_γ_ is the extended-term parameter for NaCl.^35^ The calculated Er chloride speciation data in solutions containing NaCl using the extended Debye–Hückel equation are consistent with the aqueous species derived from the HKF equation-of-state parameters in the studies of Migdisov et al.^19,20^ In the NaCl-free and NaCl-bearing Er_2_O_3_–HClO_4_ solutions, the following aqueous species were considered based on the thermodynamic modeling of the solution compositions at 25 °C: H^+^, OH^–^, Na^+^, Cl^–^, Er^3+^, ErCl^2+^, and ErCl_2_^+^. The thermodynamic data required for modeling were taken from Shock et al.^36,37^ and Migdisov et al.^38^ Following the study by Migdisov et al.,^19^ *å*_*i*_ is the ion-size parameter, with values taken from Kielland^200^ for ions of H^+^ and Cl^–^, and those for Er^3+^ and Er chloride complexes were set at 9 and 4.5 Å; *m*_*i*_ is the molar concentration; *z*_*i*_ is the charge of the *i* aqueous species. γ_*i*_ of water was calculated from the osmotic coefficient.^35^

## 4 Results and Discussion

### 4.1 UV–vis Absorbance from 25 to 75 °C

The absorbance spectra of NaCl-free and NaCl-bearing Er_2_O_3_–HClO_4_ solutions at pH 1.5 as a function of temperature are shown in Figure 1 with the experimental data listed in Table S1. At this acidity, Er hydroxyl complexes are insignificant.^11,12,38^ For the NaCl-free Er_2_O_3_–HClO_4_ solutions at 25 °C (red color line in Figure 1), there are three sharp and well-defined absorbance peaks at 379.20, 523.00, and 652.40 nm for the Er^3+^ aqua ions, which are consistent with previous studies by Carnall^39^ and Migdisov and Williams-Jones.^19^ Furthermore, comparison of this observed spectrum with the spectrum of pH of 1.5 HClO_4_ solution demonstrates that perchlorate ions do not form spectroscopically detectable complexes with Er^3+^ at 0.06 mol/kg. This result is in agreement with the earlier ion-exchange and spectroscopic studies by Choppin and Dinius^40^ and Krumholz,^41^ which demonstrated that perchlorate ions do not form complexes with trivalent REE at concentrations below 6 mol/L. No obvious absorbance peak was detected in the UV–vis spectra of pH 1.5 HClO_4_ solution at wavelengths of 350–750 nm and temperatures of 25–75 °C. Addition of NaCl to the Er_2_O_3_–HClO_4_ solutions results in a slight but systematic decrease in the UV–vis absorbance intensities of the Er^3+^ aqua ions with increasing m_Cl_/m_Er_ ratios from 0 to ∼34 (Figure 1). For instance, the absorbance spectra at 379.20 and 523.00 nm wavelengths display a decrease from ∼0.344 to 0.338 and from ∼0.197 to 0.193, respectively. The Na^+^ and Cl^–^ species in these solutions do not show relevant absorbance in the spectra of reference at these wavelength ranges.

**Figure 1:**
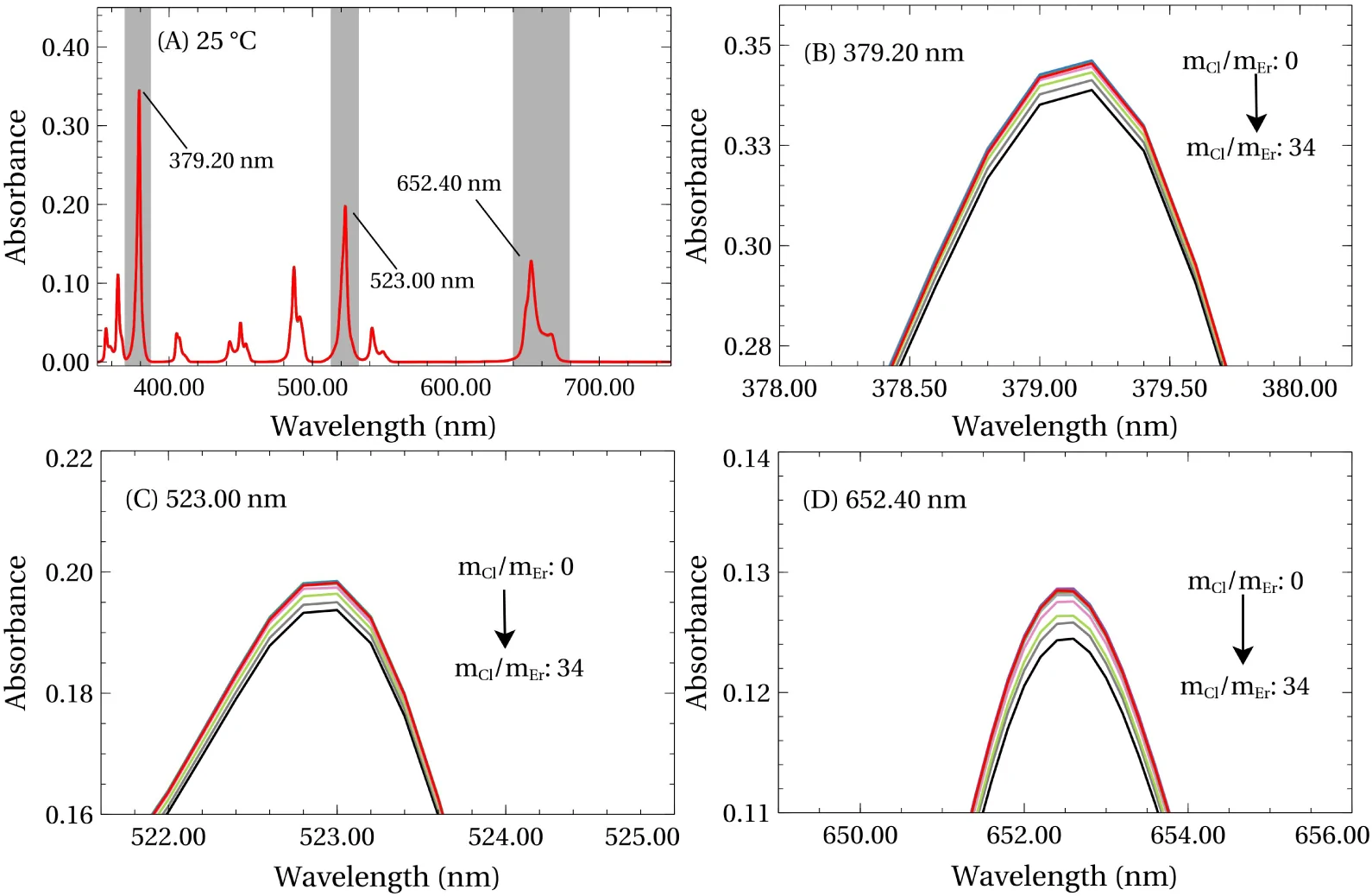
UV–vis absorbance spectra at 25 °C of solutions with pH 1.5 solutions with (A) ∼0.06 mol/kg Er and 0.2 mol/kg HClO_4_ showing the change in absorbance for major peaks at (B) 379.20 nm, (C) 523.00 nm, and (D) 652.40 nm with the addition of varying NaCl concentrations ranging from 0.0 to 1.7 mol/kg (m_Cl_/m_Er_ ranging from 0 to ∼34). All the experimental data are listed in Table S1.

A comparison of the absorbance intensities of these three major peaks as functions of temperature and m_Cl_/m_Er_ ratios is shown in Figure 2. The absorbance intensity decreases with increased temperature and displays a linear relationship with m_Cl_/m_Er_ ratios (Tables S2 and S3). For example, in an initial NaCl-free Er_2_O_3_–HClO_4_ solution (red color in Figures 1 and S1–S3), the absorbance spectrum at 379.20 nm wavelength is ∼0.344 at 25 °C and ∼0.319 at 75 °C. These results are in agreement with the UV–vis spectra observed in the REE_2_O_3_–HClO_4_ solutions at elevated temperatures in the experiments by Migdisov and Williams-Jones^19,27^ and Stepanchikova and Kolonin.^42^ Because the temperature range explored was small, no spectral shift was observed in our study under the experimental conditions. The absorbance spectrum at 379.20 nm wavelength decreases to ∼0.338 and 0.317 with increasing m_Cl_/m_Er_ ratios to 34. The same trends are observed at 523.00 and 652.40 nm wavelengths. However, due to the wavelength scale, the differences between experiments conducted at varying temperatures appear smaller compared to those at 379.20 nm. Overall, no significant spectral shift was observed across the investigated conditions, even as the m_Cl_/m_Er_ ratio increased to its highest value within the low-temperature range studied. This absence of spectral variation indicates that increasing chloride concentrations does not significantly alter the dominant Er species under these conditions. Instead, these observed decreases in absorbance suggest the expected decrease in Er^3+^ absorbance in solutions due to enhanced formation of Er chloride complexes when NaCl is added to the experimental solutions.

**Figure 2:**
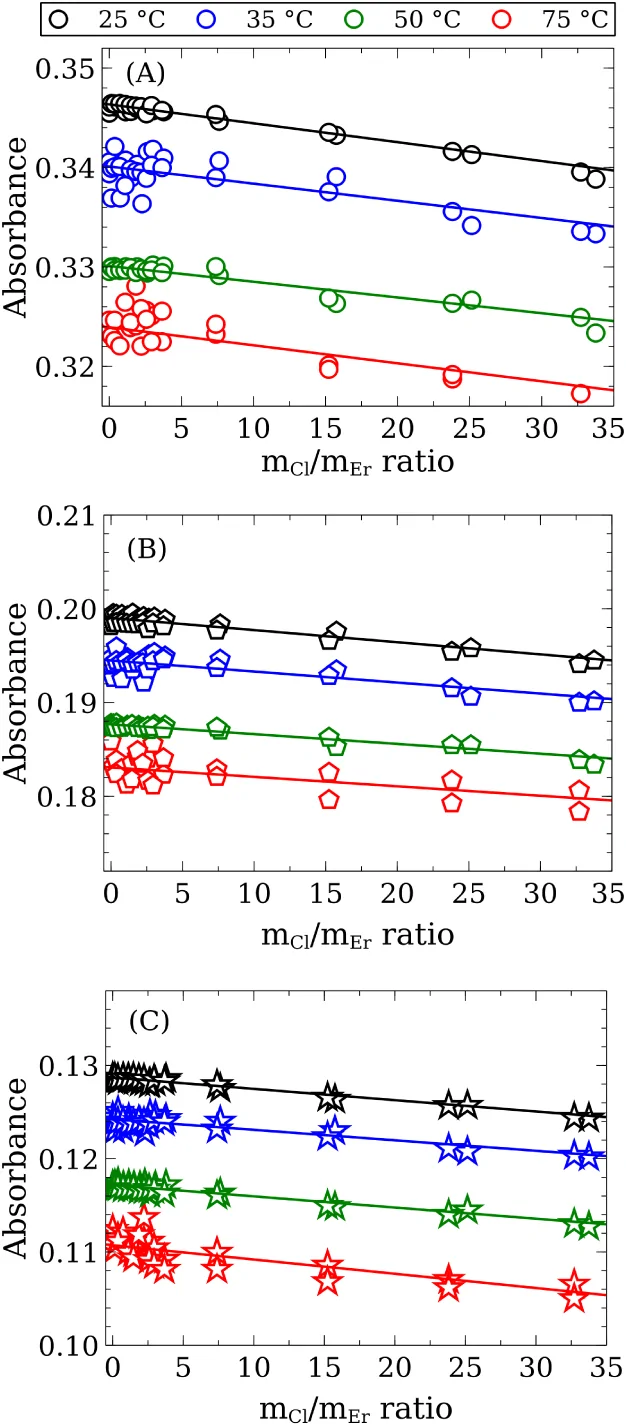
Absorbance intensities at wavelengths of (A) 379.20, (B) 523.00, and (C) 652.40 nm of UV–vis spectra as a function of the ratio of Cl to Er concentrations between 25 and 75 °C. The fitted parameters of the regressed experimental data are reported in Table S3.

The experimental uncertainty of absorbance values at 25–75 °C in this study was determined to range from 0.01 to 0.03 by measuring the UV–vis spectra of ∼0.06 mol/kg NaCl-free Er_2_O_3_–HClO_4_ solutions. The results of the rank calculations for these tolerance intervals were taken as the total number of absorbing species. The results of the rank analysis at temperatures between 25 and 75 °C (Figure 3 and Table 1) indicate that the total number of absorbing species is 2 according to a tolerance of 0.03. This suggests that there are only two absorbing Er species present at 25–75 °C under the experimental conditions investigated. Considering that the only REE species described in the literature for acidic Cl-bearing solutions are REE^3+^, REECl^2+^, and REECl_2_^+^ by Wood^43^ and the previous UV–vis study of Er chloride species at 100–250 °C by Migdisov and Williams-Jones,^19^ the absorbing species in the experimental solutions at 25–75 °C and a pH of 1.5 are interpreted to be Er^3+^ and ErCl^2+^. This interpretation is further supported by the absorbance spectra as a function of m_Cl_/m_Er_ ratios, which display a linear relationship at wavelengths of 379.20, 523.00, and 652.40 nm (Figure 2 and Table S3). The latter indicates no systematic changes in Er chloride species with increasing chloride concentration, suggesting that only ErCl^2+^ is present and not ErCl_2_^+^. These findings are in agreement with the identified species contributing to the perturbation of the water peak from *in situ* Raman spectroscopic measurements in Nd and Yb chloride solutions combined with multivariate curve resolution analysis.^44^ Furthermore, *in situ* Raman spectroscopic investigations by Smith-Schmitz et al.^45^ indicate that, under conditions of low DyCl_3_ concentrations and low temperatures, the Dy–Cl peak is predominantly attributed to the DyCl^2+^ complex.

**Figure 3:**
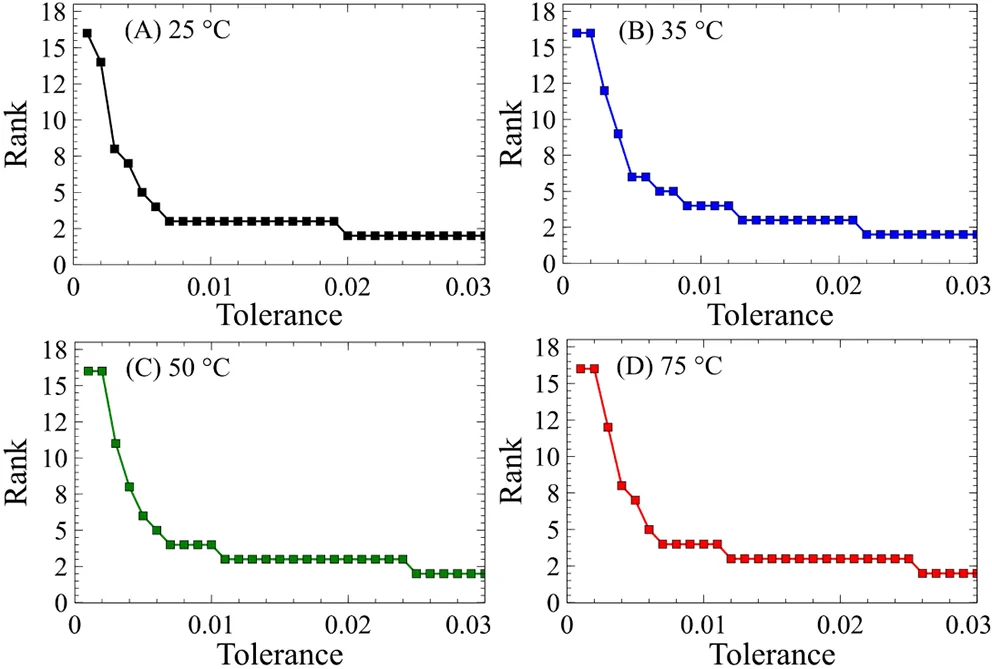
Results of rank calculations for the UV–vis experimental data at (A) 25 °C, (B) 35 °C, (C) 50 °C, and (D) 75 °C in this study for the absorbance matrix at 350–750 nm.

**Table 1:** Results of the Rank Analysis of the Absorbance Matrix at Temperatures between 25 and 75 °C^a^

*T/*°C	Wavelength/nm	Number of solutions	Rank	Absorbing species
25	350–750	45	2	Er^3+^, ErCl^2+^
35	350–750	45	2	Er^3+^, ErCl^2+^
50	350–750	45	2	Er^3+^, ErCl^2+^
75	350–750	45	2	Er^3+^, ErCl^2+^

a The rank corresponds to the number of absorbing species in the solutions.

### 4.2 Molar Absorbance Coefficient of Er^3+^ as a Function of Temperature

The molar absorbance coefficients of Er^3+^ at wavelengths of 379.20, 523.00, and 652.40 nm were calculated from the measured UV–vis spectrum of the NaCl-free Er_2_O_3_–HClO_4_ solution from 25 to 75 °C. The experimental data fits for the temperature-dependent molar absorbance coefficient are shown in Figure 4 and listed in Table 2. The molar absorbance coefficient of Er^3+^ decreases linearly from ∼5.934 to 5.491 at 379.20 nm, from ∼3.400 to 3.108 at 523.00 nm, and from ∼2.197 to 1.881 at a wavelength of 652.40 nm. The trends observed in our study are consistent with the absorbance spectra of Er-bearing solutions observed by Migdisov and Williams-Jones at higher temperatures.^19^ Fits to the experimental data indicate a linear relationship between the Er^3+^ molar absorbance coefficient and temperature with high *R* ^2^ (Table 3). The results derived from this study are the first data for the Er^3+^ molar absorbance coefficient at temperatures between 30 and 100 °C.

**Figure 4:**
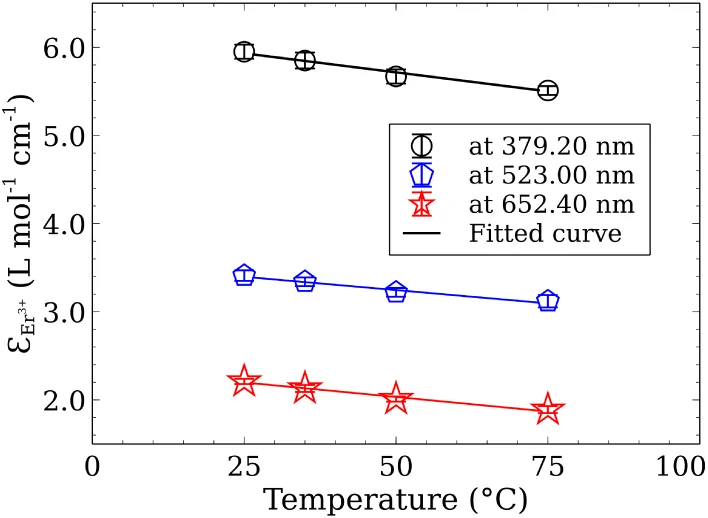
Temperature dependence of the molar absorbance coefficients (ε) of Er^3+^ at wavelengths 379.20, 523.00, and 652.40 nm and temperatures of 25–75 °C. The measured experimental data and regressions from the fitted curves are reported in Tables 2 and 3, respectively.

**Table 2:** Molar Absorbance Coefficients (ε*)* of Er^3+^ at Wavelengths 379.20, 523.00, and 652.40 nm and Temperatures of 25–75 °C^a^

*T*/°C	ε_379.20 nm_	1σ	ε_523.00 nm_	1σ	ε_652.40 nm_	1σ
25	5.95	0.08	3.41	0.06	2.21	0.03
35	5.85	0.09	3.34	0.05	2.13	0.04
50	5.67	0.08	3.22	0.05	2.01	0.03
75	5.51	0.05	3.12	0.07	1.89	0.04

a The standard deviation of the mean (1σ) is based on replicate experiments (Table S1).

**Table 3:** Molar Absorbance Coefficient of Er^3+^ Regressed from the Experimental Data (Table 2) as a Function of Temperatures between 25 and 75 °C

	Fitted equation: ε = ax + b		
Wavelength, nm	a	b	*R* ^2^
379.20	–0.009	6.155	0.982
523.00	–0.006	3.547	0.974
652.40	–0.006	2.355	0.981

### 4.3 Formation Constant for ErCl^2+^ and Comparison to Literature Data

The logarithmic values of the first formation constant (logβ_1_^0^) of the ErCl^2+^ complex (eqs 3 and 4, for *n* = 1) derived in this study are listed in Table 4 and compared to literature values (Figure 5). As the NaCl concentration increased from 0 to 1.7 mol/kg, the ionic strength of the experimental solutions increased correspondingly. To account for the effect of ionic strength on aqueous species, the ionic strength and activity coefficients of the charged species of Cl^–^, Er^3+^, and ErCl^2+^ were calculated for solutions containing 0–1.7 mol/kg NaCl at 25 and 75 °C using the extended Debye–Hückel model implemented in the GEMS code package.^46^ As shown in Figure S4, the calculated activity coefficients of Cl^–^, Er^3+^, and ErCl^2+^ are very similar over the ionic strength range corresponding to 1–1.7 mol/kg NaCl. In addition, the calculated activity coefficients at 75 °C are slightly lower than those at 25 °C across the investigated ionic strength range. These results suggest that uncertainties in activity coefficient corrections are expected to have a negligible impact on the formation constants derived between 25 and 75 °C.

**Table 4:** Comparison of the logβ_1_^0^ Values Obtained in This Study with Literature Data at Temperatures of 25–250 °C^a^ ^b^ ^c^ ^d^

	*T* (°C): 25	50	75	100	150	200	250
This study	1.06 ± 0.26^b^	1.03 ± 0.25	0.81 ± 0.20				
1				0.88 ± 0.11	1.59 ± 0.12	2.34 ± 0.11	3.09 ± 0.14
2	0.70	0.75	0.89	1.09	1.62	2.26	3.02
3	0.26						
4	0.28						
5	1.2 ± 0.5^c^						
6	0.71 ± 0.05						
7						2.17	
8	0.31			1.05	1.65	2.31	3.06
Mean	0.6 ± 0.4						

a References: 1) Migdisov and Williams-Jones;^19^ 2)­Migdisov et al.;^20^ 3) Mironov;^15^ 4) Millero;^48^ 5) Fernández-Ramírez et al.;^17^ 6) Luo and Byrne;^16^ 7) Gammons et al.;^21^ 8) Supcrt92.^47,49^

b Extrapolated from the fit in this study; see Table 5.

c Measured at 30 °C.

d The error bars represent the standard deviation (1σ) values retrieved from replicate experiments.

**Figure 5:**
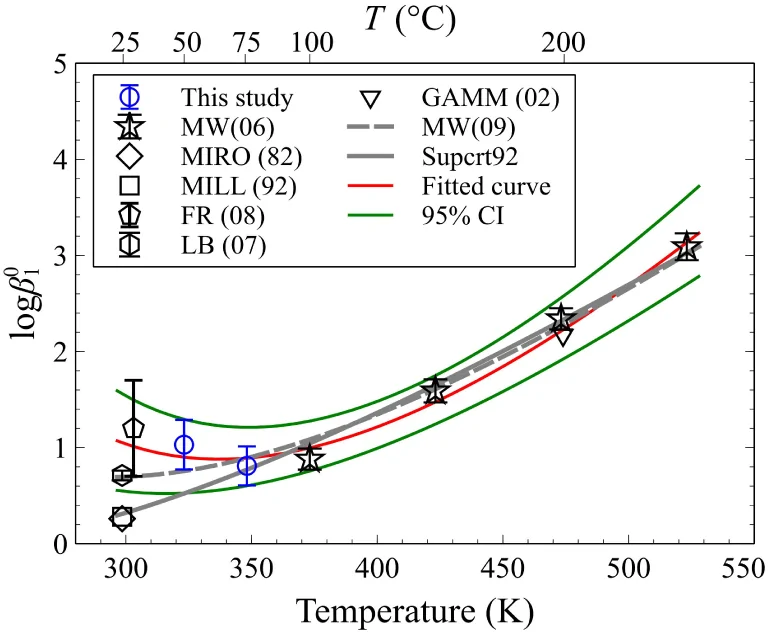
Comparison of UV–vis-derived formation constants (logβ_1_^0^) for Er chloride complexes (Er^3+^ + Cl^–^ = ErCl^2+^) with literature data over 25–250 °C (Table 4). Experimental values include 50 and 75 °C from this study and 100–250 °C from Migdisov and Williams-Jones^19^ [MW(06)]. The red line shows the regression fit (Table 5), with green lines indicating the 95% confidence interval (CI). Error bars represent 1σ from replicate experiments. Gray dashed and solid lines denote HKF fits from Migdisov and Williams-Jones^20^ [MW(09)] and predictions from the Supcrt92 database.^47,49^ Literature data: [MIRO(82)];^15^ [MILL(92)];^48^ [FR(08)];^17^ [LB(07)];^16^ [GAMM(02)].^21^

A few experimental studies have determined the formation constant of the ErCl^2+^ complex at 25 °C, with reported logβ_1_^0^ values ranging from 0.26 to 0.71 depending on the method and solution conditions.^15–17^ In contrast, studies at higher temperatures (100–250 °C), particularly those of Migdisov et al.^19,20^ and Haas et al.,^47^ consistently indicate an increase in complex stability with increasing temperature.

The logβ_1_^0^ values for the ErCl^2+^ complex derived in this study at 50 and 75 °C were therefore combined together with the UV–vis spectrophotometry data at 100–250 °C by Migdisov and Williams-Jones^19^ and fitted to a logβ_1_^0^ equation as a function of temperature from 50 to 250 °C (Figure 5). The relationship between logβ_1_^0^ and temperature is clearly nonlinear over this temperature interval, indicating that the assumption of a constant heat capacity is not valid. Consequently, the van′t Hoff equation cannot be used to derive the standard entropy and enthalpy. Instead, the fit is described by logβ_1_^0^ = *A*_0_ + *A*_1_ *T* + *A*_2_ */T* (see Supporting Materials for the derivation), where *T* is the temperature in Kelvin. The regressed parameters (*A*_0_ to *A*_3_), together with the fit coefficients (*R* ^2^ and RMSE), are listed in Table 5. The high coefficient of determination (*R* ^2^ = 0.985) indicates that the model provides an excellent representation of the combined data, despite being derived from two closely related but independently conducted experimental measurements using the same UV–vis spectrophotometric technique. Importantly, the differences between the fitted and measured logβ_1_^0^ values remain within the reported experimental uncertainties (Table S4), suggesting an internal consistency between those data. The extrapolated logβ_1_^0^ is 1.06 ± 0.26 at 25 °C, which broadly overlaps with the average logβ_1_^0^ of 0.6 ± 0.4 of all experimental data to date (Table 4). The experimental logβ_1_^0^ values for ErCl^2+^ species display a trend of a slight decrease from 25 to 75 °C, followed by an increase at higher temperatures (Figure 5). The behavior observed for the ErCl^2+^ complex in this study is consistent with trends reported for Er hydroxyl complexes in UV–vis and other REE hydroxyl complexes by UV–vis and solubility investigations.^12–14^ A similar temperature-dependent pattern has been reported for Er­(OH)^2+^ ^12^, and for La and Dy hydroxyl species, REE­(OH)^2+^.^13,14^ These studies indicate that REE-ligand complexes, including both chloride and hydroxyl species, display higher formation constants at 25 °C, followed by a decrease toward temperatures approaching the boiling point of water and subsequently a steep increase at higher temperatures, suggesting that REE-ligand complexes may exhibit temperature-dependent behavior rather than ligand-specific anomalies. The thermodynamic origin of this behavior may reflect temperature-dependent changes in enthalpy and entropy contributions to complex formation, including changes in coordination and/or solvent reorganization.^44,51,52^ However, the present experimental data do not allow direct discrimination among these potential mechanisms, which should be further investigated using molecular simulations and structural species characterization.

**Table 5:** Regressed Coefficients for the Function logβ_1_^0^= *A*_0_ + *A*_1_ *T* + *A*_2_/*T* Expressing the Logarithm of the Formation Constant for the ErCl^2+^ Complex as a Function of Temperature from 50 to 250 °^a^,^b^

	*A*_0_	*A*_1_	*A*_2_	*R* ^2^	RMSE	25 °C
logβ_1_^0^	–22.4315	0.0345	3937.9	0.985	0.146	1.06 ± 0.26

a RMSE: root-mean-square error.

b These regressions are generated using data from this study combined with data at 100–250 °C from Migdisov and Williams-Jones.^19^

In comparison with previously published data, the logβ_1_^0^ value derived in this study at 25 °C is consistent with the range reported by Luo and Byrne.^16^ Furthermore, the fitted value obtained at 30 °C shows good agreement with the experimental logβ_1_^0^ value reported by Fernández-Ramírez et al.,^17^ in which a larger uncertainty is present compared to other studies. These consistencies support the reliability of the present fitting approach and suggest that the thermodynamic trends obtained here are robust across different experimental methodologies.

A comparison of the logβ_1_^0^ values over 25–250 °C can be conducted between the results of this study and predictions from previous studies^15–17,19–21,47,48^ (Figure 5). The thermodynamic properties for REE chloride complexes were calculated by Haas et al.^47^ using the HKF semiempirical equation of state and experimental data from the literature and were compiled in the Supcrt92 database.^49^ At low temperatures (25–50 °C), the predicted values from the Supcrt92 database are lower than the derived values in this study, while the fitted values from Migdisov et al.^20^ are within the 95% confidence interval of the fitted experimental data. At temperatures between 75 and 250 °C, both the fitted values from Migdisov et al.^20^ and the predictions from the Supcrt92 database are in good agreement with the fitted experimental data in this study. Migdisov et al.^20^ calculated the thermodynamic properties for the REE chloride complexes using the logβ_1_^0^ value at 25 °C by Luo and Byrne.^16^ The discrepancies at low temperatures result from the derived values from the compilation and recalculation by Millero^48^ using the Pizer interaction model, which were used by Haas et al.^47^ Therefore, we recommend using the updated formation constants and empirical fits listed in Table 5 for more accurate modeling of the ErCl^2+^ complex between 25 and 250 °C.^50^

## 5 Conclusions

The formation constant (logβ_1_^0^) for the ErCl^2+^ complex was determined using UV–vis spectrophotometric experiments from 25 to75 °C and pH of 1.5. Rank analysis indicates that only Er^3+^ and ErCl^2+^ are likely present under the investigated conditions, even at Cl/Er concentration ratios of ∼34, suggesting the absence of the dichloride Er species (ErCl_2_^+^) at the studied low-temperature range. This finding is consistent with the results of Migdisov and Williams-Jones,^19^ who showed that although the stability of the chloride complex (ErCl^2+^) increases with temperature, the dichloride species could not be resolved below 150 °C. By integrating the logβ_1_^0^ values obtained in this study at 50 and 75 °C with high-temperature UV–vis data (100–250 °C) from Migdisov and Williams-Jones,^19^ a continuous fit was established, allowing extrapolation to 25 °C. The extrapolated logβ_1_^0^ value of 1.06 ± 0.26 at 25 °C suggests a decrease in ErCl^2+^ stability from 25 to 75 °C followed by a marked increase at higher temperatures. Notably, this behavior mirrors the trend observed for the Er­(OH)^2+^ species by Han and Gysi,^12^ suggesting that such temperature-dependent stability patterns may represent a previously unrecognized general feature of REE-ligand complexation rather than a ligand-species anomaly. Overall, the empirical fit derived from the UV–vis experiments in this study and by Migdisov and Williams-Jones^19^ provides a coherent description of REE chloride speciation across a broad temperature range. These results provide important insights into natural systems, particularly in the formation of heavy and light REE ion-adsorption deposits at ambient temperature.^53^ The REECl^2+^ complex is found to be more stable than previously predicted, indicating a stronger role for chloride complexation in controlling REE mobility under low-pH conditions. Consequently, REE transport is enhanced in acidic environments, whereas increasing pH at low temperatures promotes deposition, with implications for REE fractionation and resource formation.

## Supplementary Material



## Data Availability

Data are available through Mendeley Data at https://doi.org/10.17632/2cg5mxhn77.1. The preliminary data set doi can be accessed here: https://data.mendeley.com/preview/2cg5mxhn77?a=bf3ac243-c41a-427d-8232-4534cd6845a1

## Notes

A brief description of the relation between thermodynamic properties and the fitted parameters of formation constants; UV–vis absorbance spectra of solutions at 35 °C (Figure S1), 50 °C (Figure S2), and 75 °C (Figure S3); calculated ionic strengths and activity coefficients of Cl^–^, Er^3+^, and ErCl^2+^ in solutions containing 0–1.7 mol/kg NaCl at 25 and 75 °C (Figure S4); experimental details of ratios of Cl to Er concentrations and measured UV–vis spectrophotometric absorbance at selected wavelengths (Table S1); mean and standard deviation of determined UV–vis absorbance at selected wavelengths (Table S2); fitted parameters of determined UV–vis absorbance as a function of the ratio of Cl to Er concentration (Table S3); differences between the fitted model and measured experimental values in this study, data fitted from the experimental literature, and values from Supcrt92 (Table S4) (PDF)
