# Supplementary material for: Spectrophotometric Study of Erbium(III) Speciation in Chloride Solutions from 25 to 75 °C
Source: ACS Earth Space Chem. 2026 Aug 31;10(9):2053–61. doi: 10.1021/acsearthspacechem.6c00161 (PMC13592072; doi:10.1021/acsearthspacechem.6c00161)
Supplement: Supplementary file 1 [file sp6c00161_si_001.pdf]

Spectrophotometric study of erbium (III) speciation in chloride solutions from 25 to 75 °C

Hannah Juan Han[1]*, Alexander P. Gysi[1,2], Nicole C. Hurtig[1,2], Artas A. Migdisov[3]

[1]New Mexico Bureau of Geology and Mineral Resources, New Mexico Institute of Mining and Technology, 801 Leroy Place, Socorro, NM 87801, USA
[2]Department of Earth and Environmental Science, New Mexico Institute of Mining and Technology, 801 Leroy Place, Socorro, NM 87801, USA
[3]McGill University, Earth&Environ. Sciences, Montréal H3A 0G4, Canada.

* Corresponding author: hannah.juan.han@nmt.edu

Keywords: Rare earth elements, UV-vis spectrophotometry, Erbium, Chloride, Formation constant, Speciation

submitted to ACS Earth and Space Chemistry

REVISION 1

Relationship between enthalpy, entropy, and the fitted parameters of formation constants

The standard enthalpy change at a constant pressure was obtained by applying the van't Hoff equation to the temperature dependence of the equilibrium constants,

$$\frac{d(\ln K)}{dT} = \frac{\Delta H^0}{RT^2}$$

The heat capacity at a constant pressure was determined using Kirchhoff's equation,

$$\left[\frac{d\Delta H}{dT}\right]_P = \Delta C_p$$

where K is the equilibrium constant, T is the temperature in Kelvin, R is the ideal gas constant. ΔC_p was described using the equation of Haas and Fisher[51],

$$\Delta C_p = a_0 + a_1 T + a_2 T^{-2} + a_3 T^{-0.5} + a_4 T^2$$

The ΔH^0 was obtained by integrating the ΔC_p equation with a constant enthalpy term h_{Tr},

$$\Delta H^0 = h_{Tr} + a_0 T + (a_1/2)T^2 - a_2/T + 2a_3 T^{0.5} + (a_4/3)T^3$$

where h_{Tr} is defined as,

$$h_{Tr} = \Delta H^0 - a_0 T_r - (a_1/2)T_r^2 + a_2/T_r - 2a_3 T^{0.5} - (a_4/3){T_r}^3$$

The integration of the van't Hoff yields the following four-parameter equation,

$$\ln K = A + BT + C/T + D\ln(T)$$

where A−C are parameters corresponding to the coefficients A_0–A_2 in this study,

$$A = A_0 \ln(10) = (S_{Tr} - a_0)/R$$
$$B = A_1 \ln(10) = a_1/(2R)$$

$$C = A_2 \ln(10) = -h_{Tr}/R$$

And entropy can be retrieved from the constant entropy term (S_{Tr}),

$$s_{Tr} = \Delta S^0 - a_0 \ln T_r - a_1 T_r + a_2/\left(2{T_r}^2\right) + 2a_3 {T_r}^{-0.5} - (a_4/2){T_r}^2$$

where the coefficients of a_0, a_1, a_2, a_3, and a_4 can be obtained by conducting different isotherms' experiments.

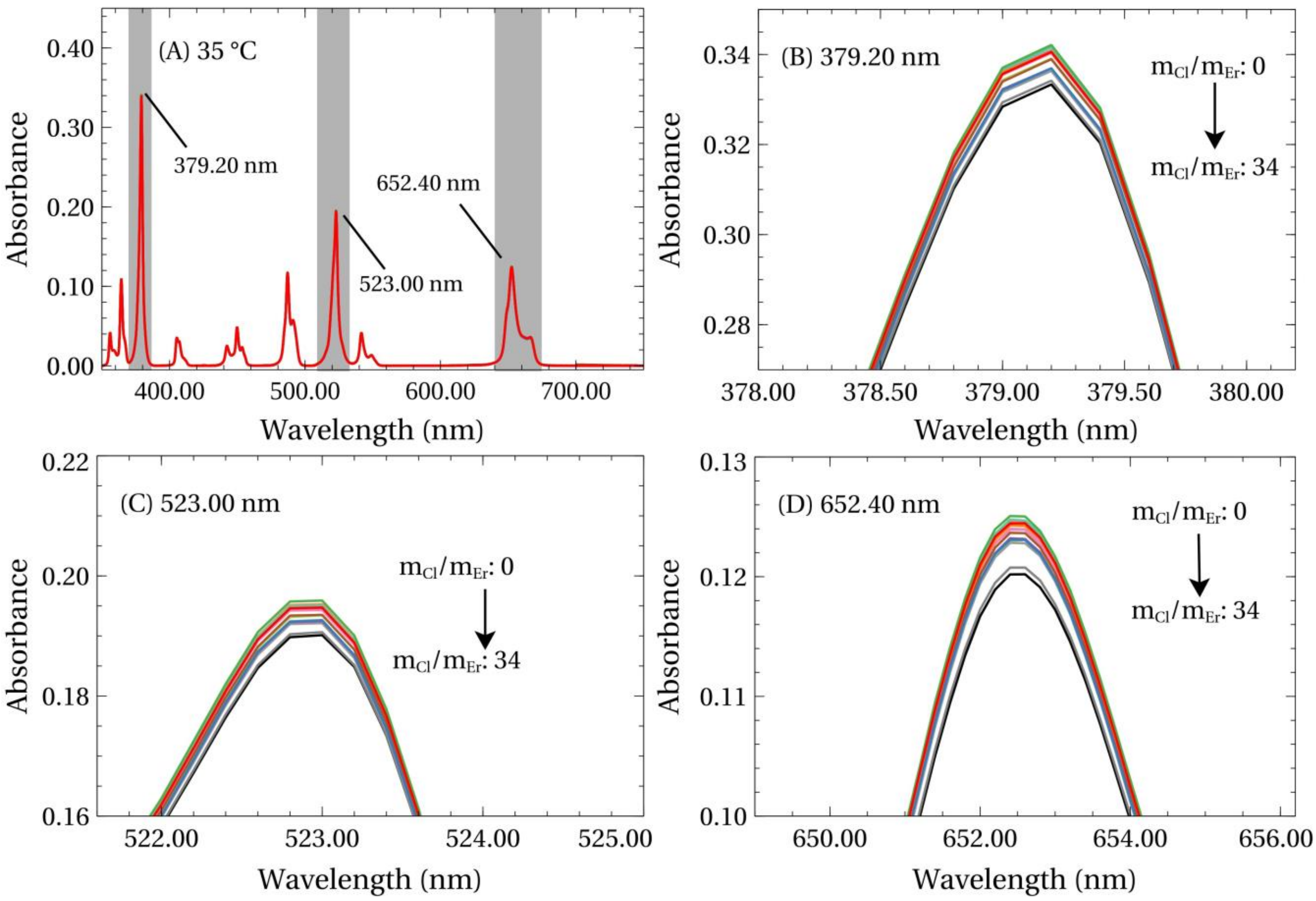

Figure S1. UV-vis absorbance spectra at 35 °C of solutions with pH 1.5 solutions with (A) ~0.06 mol/kg Er, and 0.2 mol/kg $HClO_4$ showing the change in absorbance for major peaks at (B) 379.20 nm, (C) 523.00 nm, and (D) 652.40 nm with the addition of varying NaCl concentrations ranging from 0.0 to 1.7 mol/kg (m_{Cl}/m_{Er} ranging from 0 to ~34). All the experimental data are listed in Table S1.

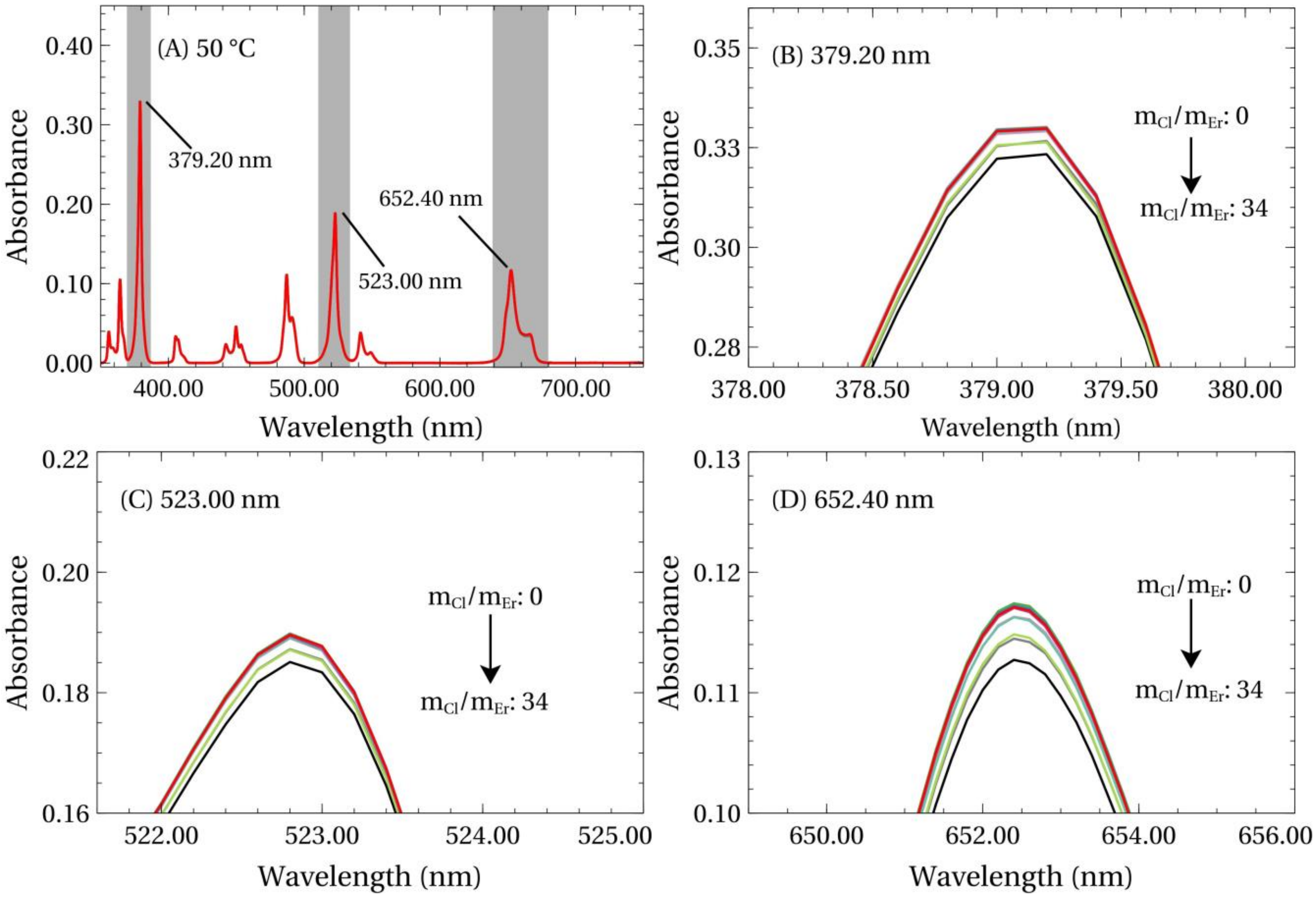

Figure S2. UV-vis absorbance spectra at 50 °C of solutions with pH 1.5 solutions with (A) ~0.06 mol/kg Er, and 0.2 mol/kg $HClO_4$ showing the change in absorbance for major peaks at (B) 379.20 nm, (C) 523.00 nm, and (D) 652.40 nm with the addition of varying NaCl concentrations ranging from 0.0 to 1.7 mol/kg (m_{Cl}/m_{Er} ranging from 0 to ~34). All the experimental data are listed in Table S1.

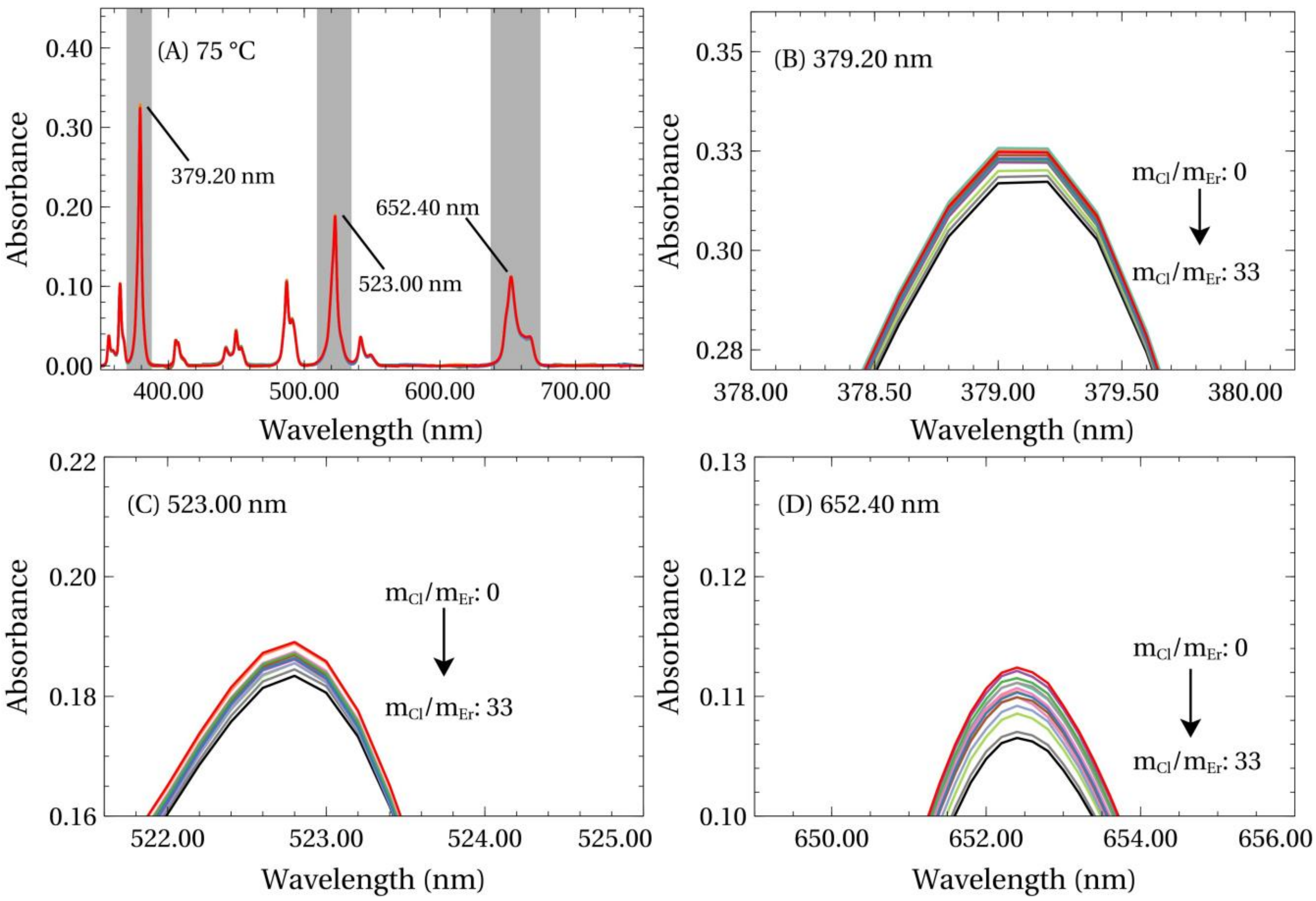

Figure S3. UV-vis absorbance spectra at 75 °C of solutions with pH 1.5 solutions with (A) ~0.06 mol/kg Er, and 0.2 mol/kg $HClO_4$ showing the change in absorbance for major peaks at (B) 379.20 nm, (C) 523.00 nm, and (D) 652.40 nm with the addition of varying NaCl concentrations ranging from 0.0 to 1.7 mol/kg (m_{Cl}/m_{Er} ranging from 0 to ~33). All the experimental data are listed in Table S1.

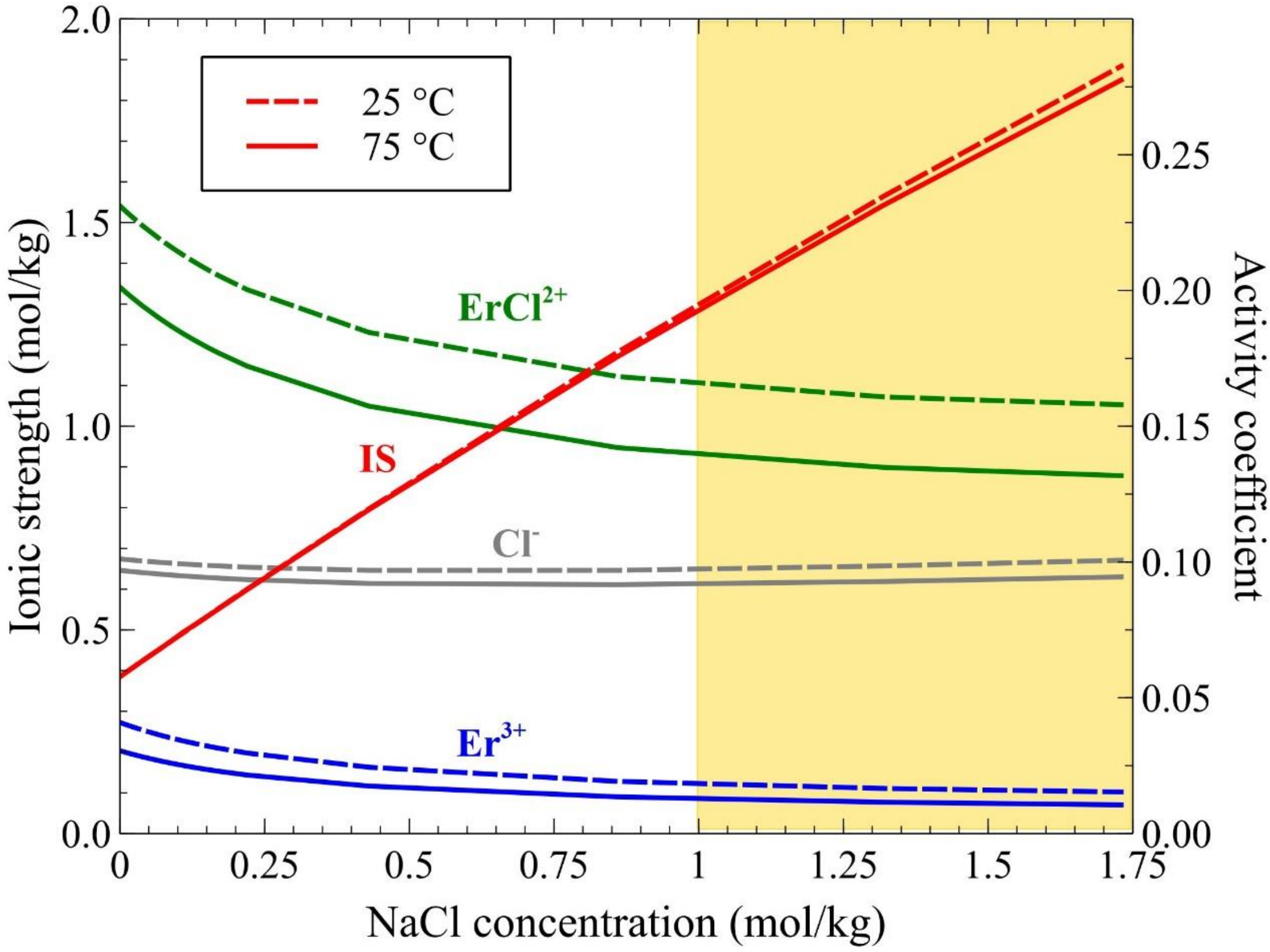

Figure S4. Calculated ionic strength (IS) and activity coefficients of the charged aqueous species Cl^-, Er^{3+}, and $ErCl^{2+}$ as a function of NaCl concentration (0−1.7 mol/kg) at 25 and 75 °C using the extended Debye-Hückel model implemented in the GEMS code package[47]. The yellow shaded region highlights the ionic strength range corresponding to 1.0−1.7 mol/kg NaCl.

Table S1. Compositions of the experimental solutions and measured absorbance (*A*) from UV-vis experiments of ~0.06 mol/kg Er in pH of 1.5 of 0.2 mol/kg $HClO_4$ solutions with varying NaCl concentrations at temperatures from 25 to 75 °C.

Sample	mEr	mNaCl	m_{Cl}/m_{Er}		*A*	
	mol/kg	mol/kg		379.20 nm	523.00nm	652.40nm
T= 25 °C						
Er_a_1	0.06	0.00	0.00	0.345	0.199	0.128
Er_a_2	0.06	0.01	0.15	0.346	0.199	0.128
Er_a_3	0.06	0.02	0.38	0.346	0.199	0.128
Er_a_4	0.06	0.04	0.76	0.346	0.199	0.129
Er_a_5	0.06	0.07	1.14	0.346	0.199	0.129
Er_a_6	0.06	0.09	1.51	0.346	0.199	0.128
Er_a_7	0.06	0.11	1.94	0.346	0.199	0.128
Er_a_8	0.06	0.13	2.27	0.346	0.199	0.128
Er_a_9	0.06	0.15	2.64	0.346	0.199	0.128
Er_a_10	0.06	0.17	3.03	0.346	0.199	0.128
Er_a_11	0.06	0.22	3.78	0.346	0.199	0.129
Er_a_12	0.06	0.43	7.62	0.345	0.198	0.128
Er_a_13	0.05	0.86	15.75	0.343	0.198	0.126
Er_a_14	0.05	1.32	25.14	0.341	0.196	0.126
Er_a_15	0.05	1.73	33.75	0.339	0.194	0.124
Er_b_1	0.06	0.00	0.00	0.346	0.198	0.129
Er_b_2	0.06	0.01	0.15	0.346	0.199	0.129
Er_b_3	0.06	0.02	0.37	0.346	0.198	0.129
Er_b_4	0.06	0.04	0.73	0.346	0.198	0.129
Er_b_5	0.06	0.06	1.10	0.346	0.198	0.129
Er_b_6	0.06	0.09	1.46	0.346	0.198	0.129
Er_b_7	0.06	0.11	1.85	0.346	0.198	0.129
Er_b_8	0.06	0.13	2.21	0.346	0.198	0.129

Er_b_9	0.06	0.15	2.58	0.345	0.198	0.128
Er_b_10	0.06	0.17	2.93	0.346	0.198	0.128
Er_b_11	0.06	0.21	3.66	0.346	0.198	0.128
Er_b_12	0.06	0.43	7.39	0.345	0.198	0.128
Er_b_13	0.06	0.86	15.23	0.344	0.197	0.127
Er_b_14	0.05	1.29	23.81	0.342	0.195	0.126
Er_b_15	0.05	1.72	32.71	0.340	0.194	0.124
Er_c_1	0.06	0.00	0.00	0.340	0.195	0.126
Er_c_2	0.06	0.01	0.16	0.340	0.195	0.126
Er_c_3	0.06	0.02	0.38	0.340	0.195	0.126
Er_c_4	0.06	0.04	0.76	0.340	0.195	0.126
Er_c_5	0.06	0.07	1.15	0.340	0.195	0.126
Er_c_6	0.06	0.09	1.53	0.339	0.194	0.126
Er_c_7	0.06	0.11	1.96	0.341	0.195	0.126
Er_c_8	0.06	0.13	2.31	0.340	0.195	0.126
Er_c_9	0.06	0.15	2.68	0.340	0.195	0.126
Er_c_10	0.06	0.17	3.07	0.340	0.195	0.126
Er_c_11	0.06	0.22	3.83	0.340	0.195	0.126
Er_c_12	0.06	0.43	7.77	0.339	0.194	0.125
Er_c_13	0.05	0.86	15.93	0.338	0.193	0.125
Er_c_14	0.05	1.32	25.02	0.336	0.192	0.123
Er_c_15	0.05	1.73	33.50	0.334	0.190	0.122
T= 35 °C						
Er_a_1	0.06	0.00	0.00	0.341	0.195	0.124
Er_a_2	0.06	0.01	0.15	0.337	0.193	0.123
Er_a_3	0.06	0.02	0.38	0.342	0.196	0.125
Er_a_4	0.06	0.04	0.76	0.337	0.192	0.123
Er_a_5	0.06	0.07	1.14	0.341	0.195	0.124

Er_a_6	0.06	0.09	1.51	0.339	0.194	0.124
Er_a_7	0.06	0.11	1.94	0.340	0.194	0.124
Er_a_8	0.06	0.13	2.27	0.336	0.192	0.123
Er_a_9	0.06	0.15	2.64	0.342	0.195	0.125
Er_a_10	0.06	0.17	3.03	0.342	0.195	0.125
Er_a_11	0.06	0.22	3.78	0.341	0.195	0.124
Er_a_12	0.06	0.43	7.62	0.341	0.195	0.124
Er_a_13	0.05	0.86	15.75	0.339	0.193	0.123
Er_a_14	0.05	1.32	25.14	0.334	0.191	0.121
Er_a_15	0.05	1.73	33.75	0.333	0.190	0.120
Er_b_1	0.06	0.00	0.00	0.339	0.194	0.124
Er_b_2	0.06	0.01	0.15	0.340	0.194	0.124
Er_b_3	0.06	0.02	0.37	0.340	0.194	0.124
Er_b_4	0.06	0.04	0.73	0.340	0.194	0.124
Er_b_5	0.06	0.06	1.10	0.338	0.194	0.124
Er_b_6	0.06	0.09	1.46	0.340	0.194	0.124
Er_b_7	0.06	0.11	1.85	0.340	0.194	0.124
Er_b_8	0.06	0.13	2.21	0.340	0.194	0.124
Er_b_9	0.06	0.15	2.58	0.339	0.194	0.124
Er_b_10	0.06	0.17	2.93	0.340	0.194	0.124
Er_b_11	0.06	0.21	3.66	0.340	0.195	0.124
Er_b_12	0.06	0.43	7.39	0.339	0.194	0.123
Er_b_13	0.06	0.86	15.23	0.338	0.193	0.122
Er_b_14	0.05	1.29	23.81	0.336	0.192	0.121
Er_b_15	0.05	1.72	32.71	0.334	0.190	0.120
Er_c_1	0.06	0.00	0.00	0.334	0.191	0.121
Er_c_2	0.06	0.01	0.16	0.334	0.191	0.122
Er_c_3	0.06	0.02	0.38	0.334	0.191	0.121

Er_c_4	0.06	0.04	0.76	0.334	0.191	0.121
Er_c_5	0.06	0.07	1.15	0.334	0.191	0.121
Er_c_6	0.06	0.09	1.53	0.334	0.190	0.121
Er_c_7	0.06	0.11	1.96	0.334	0.191	0.121
Er_c_8	0.06	0.13	2.31	0.334	0.191	0.121
Er_c_9	0.06	0.15	2.68	0.334	0.190	0.121
Er_c_10	0.06	0.17	3.07	0.334	0.191	0.121
Er_c_11	0.06	0.22	3.83	0.334	0.191	0.121
Er_c_12	0.06	0.43	7.77	0.333	0.190	0.120
Er_c_13	0.05	0.86	15.93	0.332	0.189	0.119
Er_c_14	0.05	1.32	25.02	0.330	0.188	0.118
Er_c_15	0.05	1.73	33.50	0.328	0.187	0.117
T= 50 °C						
Er_a_1	0.06	0.00	0.00	0.330	0.188	0.117
Er_a_2	0.06	0.01	0.15	0.330	0.188	0.117
Er_a_3	0.06	0.02	0.38	0.330	0.188	0.117
Er_a_4	0.06	0.04	0.76	0.330	0.187	0.117
Er_a_5	0.06	0.07	1.14	0.330	0.188	0.117
Er_a_6	0.06	0.09	1.51	0.330	0.188	0.117
Er_a_7	0.06	0.11	1.94	0.330	0.188	0.117
Er_a_8	0.06	0.13	2.27	0.330	0.187	0.117
Er_a_9	0.06	0.15	2.64	0.329	0.187	0.116
Er_a_10	0.06	0.17	3.03	0.330	0.188	0.117
Er_a_11	0.06	0.22	3.78	0.330	0.188	0.117
Er_a_12	0.06	0.43	7.62	0.329	0.187	0.116
Er_a_13	0.05	0.86	15.75	0.326	0.185	0.115
Er_a_14	0.05	1.32	25.14	0.327	0.185	0.114
Er_a_15	0.05	1.73	33.75	0.323	0.183	0.113
Er_b_1	0.06	0.00	0.00	0.330	0.187	0.117
Er_b_2	0.06	0.01	0.15	0.330	0.187	0.117

Er_b_3	0.06	0.02	0.37	0.330	0.187	0.117
Er_b_4	0.06	0.04	0.73	0.330	0.187	0.117
Er_b_5	0.06	0.06	1.10	0.330	0.187	0.117
Er_b_6	0.06	0.09	1.46	0.330	0.188	0.117
Er_b_7	0.06	0.11	1.85	0.329	0.187	0.117
Er_b_8	0.06	0.13	2.21	0.330	0.187	0.117
Er_b_9	0.06	0.15	2.58	0.330	0.187	0.117
Er_b_10	0.06	0.17	2.93	0.330	0.187	0.117
Er_b_11	0.06	0.21	3.66	0.329	0.187	0.116
Er_b_12	0.06	0.43	7.39	0.330	0.187	0.116
Er_b_13	0.06	0.86	15.23	0.327	0.186	0.115
Er_b_14	0.05	1.29	23.81	0.326	0.185	0.114
Er_b_15	0.05	1.72	32.71	0.325	0.184	0.113
Er_c_1	0.06	0.00	0.00	0.324	0.184	0.115
Er_c_2	0.06	0.01	0.16	0.325	0.184	0.115
Er_c_3	0.06	0.02	0.38	0.325	0.184	0.115
Er_c_4	0.06	0.04	0.76	0.325	0.184	0.115
Er_c_5	0.06	0.07	1.15	0.325	0.184	0.115
Er_c_6	0.06	0.09	1.53	0.325	0.184	0.115
Er_c_7	0.06	0.11	0.20	0.324	0.184	0.115
Er_c_8	0.06	0.13	2.31	0.325	0.185	0.115
Er_c_9	0.06	0.15	2.68	0.325	0.184	0.115
Er_c_10	0.06	0.17	3.07	0.325	0.184	0.115
Er_c_11	0.06	0.22	3.83	0.324	0.184	0.115
Er_c_12	0.06	0.43	7.77	0.324	0.184	0.114
Er_c_13	0.05	0.86	15.93	0.323	0.183	0.113
Er_c_14	0.05	1.32	25.02	0.321	0.180	0.112
Er_c_15	0.05	1.73	33.50	0.319	0.181	0.111
T= 75 °C						

Er_a_1	0.06	0.00	0.00	0.325	0.186	0.112
Er_a_2	0.06	0.01	0.15	0.323	0.183	0.110
Er_a_3	0.06	0.02	0.37	0.323	0.184	0.112
Er_a_4	0.06	0.04	0.73	0.322	0.183	0.112
Er_a_5	0.06	0.06	1.10	0.329	0.187	0.113
Er_a_5	0.06	0.09	1.46	0.324	0.184	0.110
Er_a_6	0.06	0.11	1.85	0.324	0.184	0.111
Er_a_7	0.06	0.13	2.21	0.322	0.184	0.111
Er_a_8	0.06	0.15	2.58	0.326	0.184	0.111
Er_a_9	0.06	0.17	2.93	0.325	0.186	0.110
Er_a_10	0.06	0.21	3.66	0.322	0.182	0.109
Er_a_11	0.06	0.43	7.39	0.323	0.183	0.110
Er_a_12	0.06	0.86	15.23	0.320	0.183	0.109
Er_a_13	0.05	1.29	23.81	0.319	0.182	0.107
Er_a_14	0.05	1.72	32.71	0.317	0.181	0.107
Er_b_1	0.06	0.00	0.00	0.317	0.178	0.109
Er_b_2	0.06	0.01	0.15	0.319	0.182	0.111
Er_b_3	0.06	0.02	0.37	0.325	0.182	0.111
Er_b_4	0.06	0.04	0.73	0.323	0.180	0.109
Er_b_5	0.06	0.06	1.10	0.327	0.181	0.110
Er_b_6	0.06	0.09	1.46	0.325	0.182	0.109
Er_b_7	0.06	0.11	1.85	0.328	0.185	0.112
Er_b_8	0.06	0.13	2.21	0.326	0.183	0.114
Er_b_9	0.06	0.15	2.58	0.325	0.182	0.109
Er_b_10	0.06	0.17	2.93	0.323	0.181	0.109
Er_b_11	0.06	0.21	3.66	0.326	0.184	0.108
Er_b_12	0.06	0.43	7.39	0.324	0.182	0.108
Er_b_13	0.06	0.86	15.23	0.320	0.180	0.107

Er_b_14	0.05	1.29	23.81	0.319	0.179	0.106
Er_b_15	0.05	1.72	32.71	0.317	0.178	0.105
Er_c_1	0.06	0.00	0.00	0.315	0.177	0.108
Er_c_2	0.06	0.01	0.16	0.309	0.175	0.107
Er_c_3	0.06	0.02	0.38	0.317	0.179	0.109
Er_c_4	0.06	0.04	0.76	0.318	0.179	0.108
Er_c_5	0.06	0.07	1.15	0.317	0.179	0.109
Er_c_6	0.06	0.09	1.53	0.315	0.178	0.106
Er_c_7	0.06	0.11	1.96	0.320	0.180	0.109
Er_c_8	0.06	0.13	2.31	0.315	0.178	0.106
Er_c_9	0.06	0.15	2.68	0.316	0.178	0.107
Er_c_10	0.06	0.17	3.07	0.315	0.178	0.106
Er_c_11	0.06	0.22	3.83	0.316	0.177	0.107
Er_c_12	0.06	0.43	7.77	0.316	0.178	0.106
Er_c_13	0.05	0.86	15.93	0.317	0.179	0.106
Er_c_14	0.05	1.32	25.02	0.316	0.176	0.105
Er_c_15	0.05	1.73	33.50	0.316	0.175	0.107

Table S2. Composition of ~0.06 mol/kg Er in pH of 1.5 of 0.2 mol/kg $HClO_4$ solutions with varying NaCl concentrations at temperatures from 25 to 75 °C showing ratios of Cl to Er total concentrations (m_{Cl}/m_{Er}) and absorbance (A) at selected wavelengths 379.20, 523.00, and 652.40 nm. The standard deviation of the mean (1σ) is based on replicate experiments (Table S1).

m_{Cl}/m_{Er}	379.20 nm		523.00 nm		652.40 nm	
	A	1σ	*A*	1σ	*A*	1σ
T=25 °C						
0.00	0.344	0.003	0.197	0.002	0.128	0.001
0.15	0.344	0.004	0.198	0.003	0.128	0.002
0.38	0.344	0.003	0.198	0.002	0.128	0.001
0.75	0.344	0.003	0.198	0.002	0.128	0.001
1.13	0.344	0.003	0.198	0.002	0.128	0.001
1.50	0.344	0.004	0.197	0.003	0.128	0.002
1.92	0.344	0.003	0.197	0.002	0.128	0.001
2.27	0.344	0.003	0.197	0.002	0.128	0.001
2.63	0.344	0.003	0.197	0.002	0.127	0.001
3.01	0.344	0.003	0.197	0.002	0.128	0.001
3.76	0.344	0.003	0.197	0.002	0.128	0.001
7.60	0.343	0.003	0.197	0.002	0.127	0.001
15.64	0.341	0.003	0.196	0.002	0.126	0.001
24.66	0.339	0.003	0.194	0.002	0.125	0.001
33.32	0.337	0.003	0.193	0.002	0.124	0.001
T=35 °C						
0.00	0.338	0.004	0.193	0.002	0.123	0.002
0.15	0.337	0.003	0.192	0.002	0.123	0.001
0.38	0.339	0.004	0.194	0.002	0.124	0.002
0.75	0.337	0.003	0.192	0.002	0.123	0.001
1.13	0.338	0.003	0.193	0.002	0.123	0.002
1.50	0.337	0.003	0.193	0.002	0.123	0.002
1.92	0.338	0.004	0.193	0.002	0.123	0.002
2.27	0.337	0.003	0.192	0.002	0.123	0.001
2.63	0.338	0.004	0.193	0.002	0.123	0.002
3.01	0.339	0.004	0.194	0.002	0.123	0.002

3.76	0.338	0.004	0.193	0.002	0.123	0.002
7.60	0.338	0.004	0.193	0.002	0.122	0.002
15.64	0.336	0.004	0.192	0.002	0.122	0.002
24.66	0.333	0.003	0.190	0.002	0.120	0.002
33.32	0.332	0.003	0.189	0.002	0.119	0.002
T=50 °C						
0.00	0.328	0.003	0.186	0.002	0.116	0.001
0.15	0.328	0.003	0.186	0.002	0.116	0.001
0.38	0.328	0.003	0.186	0.002	0.116	0.001
0.75	0.328	0.003	0.186	0.002	0.116	0.001
1.13	0.328	0.003	0.186	0.002	0.116	0.001
1.50	0.328	0.003	0.186	0.002	0.116	0.001
1.33	0.328	0.003	0.186	0.002	0.116	0.001
2.27	0.328	0.003	0.186	0.002	0.116	0.001
2.63	0.328	0.003	0.186	0.002	0.116	0.001
3.01	0.328	0.003	0.186	0.002	0.116	0.001
3.76	0.328	0.003	0.186	0.002	0.116	0.001
7.60	0.328	0.003	0.186	0.002	0.115	0.001
15.64	0.325	0.002	0.185	0.002	0.114	0.001
24.66	0.325	0.003	0.184	0.003	0.113	0.001
33.32	0.323	0.003	0.183	0.002	0.112	0.001
T=75 °C						
0.00	0.319	0.005	0.180	0.005	0.110	0.002
0.15	0.317	0.007	0.180	0.005	0.109	0.002
0.37	0.322	0.004	0.182	0.002	0.111	0.001
0.74	0.321	0.002	0.181	0.002	0.110	0.002
1.12	0.324	0.006	0.183	0.004	0.111	0.002
1.49	0.321	0.005	0.181	0.003	0.109	0.002
1.89	0.324	0.004	0.183	0.003	0.110	0.001
2.25	0.321	0.006	0.182	0.003	0.110	0.004
2.61	0.322	0.005	0.181	0.003	0.109	0.002
2.98	0.321	0.005	0.182	0.004	0.108	0.002

3.72	0.321	0.005	0.181	0.003	0.108	0.001
7.52	0.321	0.005	0.181	0.003	0.108	0.002
15.46	0.319	0.001	0.180	0.002	0.107	0.001
24.21	0.318	0.002	0.179	0.003	0.106	0.001
32.97	0.317	0.001	0.178	0.003	0.106	0.001

Table S3. Regressed fits of the measured absorbance of Er^{3+} at wavelengths of 379.20, 523.00, And 652.40 nm as function of ratio of Cl to Er concentration (m_{Cl}/m_{Er}) between 25 and 75 °C.

Wavelength/nm	*T*/°C	Fitted equation: *A*=ax+b		R^2
		a x 10^3	b	
379.20	25	−0.206	0.346	0.977
	35	−0.186	0.340	0.607
	50	−0.170	0.330	0.922
	75	−0.237	0.325	0.722
523.00	25	−0.137	0.199	0.898
	35	−0.124	0.194	0.669
	50	−0.109	0.188	0.933
	75	−0.129	0.184	0.496
652.40	25	−0.127	0.129	0.980
	35	−0.118	0.124	0.835
	50	−0.126	0.117	0.958
	75	−0.173	0.111	0.671

Table S4. Comparison of the calculated $\log\beta_1^0$ values obtained from the fitted model over the temperature range of 50–250 °C using the regressed parameters listed in Table 5, with experimentally measured values, data fitted by Migdisov et al.[19] and values from Supcrt92[48,50]. The fitted model reproduces the experimental data within the reported experimental uncertainty limits.

T/°C	Fitted model	Measured	Δ[a]	Migdisov et al.	Δ[b]	Supct92	Δ[c]
25	1.06			0.70	0.36	0.31	0.75
50	0.90	1.03	−0.13	0.75	0.15	0.52	0.38
75	0.89	0.81	0.08	0.89	0.00	0.77	0.12

[a] Difference: fitted model−measured experimental data;
[b] Difference: fitted model−Migdisov et al.[19];
[c] Difference: fitted model−Supcrt92[48,50];
